# Supplementary material for: TOTUM-854 Human Circulating Bioactives Preserve Endothelial Cell Function
Source: Nutrients. 2025 Apr 11;17(8):1331. doi: 10.3390/nu17081331 (PMC12030166; doi:10.3390/nu17081331)
Supplement: Supplementary file 1 [file nutrients-17-01331-s001.zip › nutrients-3476813-supplementary.pdf]

Oleuropein glucuronide (2 isomers)	Hydroxytyrosol sulfate isomer
MRM transition: 553→377	MRM transition: 233→153
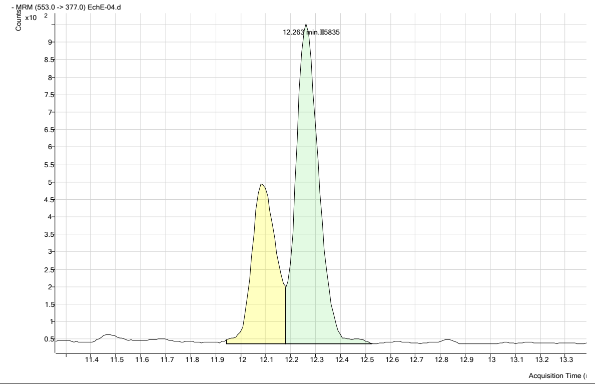	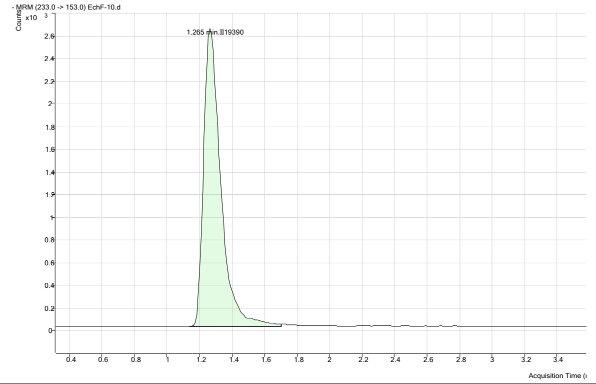
**Luteolin glucuronides (3 isomers)**	**Homovanillic acid sulfate isomer**
MRM transition: 461→285	MRM transition: 261→181
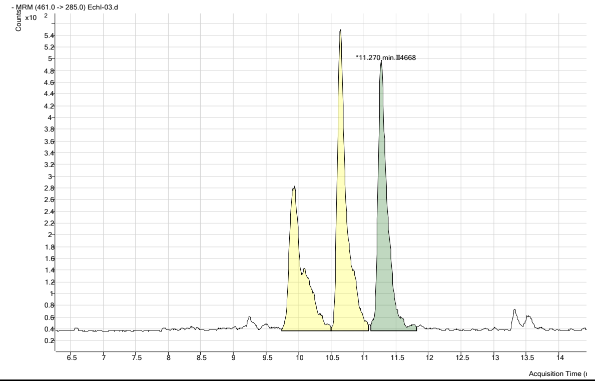	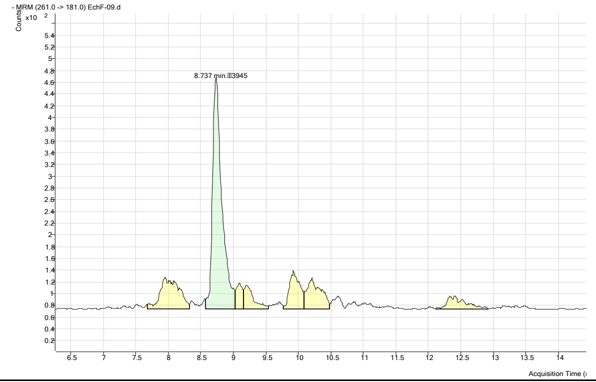
**Dihydrocaffeic acid sulfate isomer**	**Ferulic acid sulfate isomer**
MRM transition: 261→181	MRM transition: 273→193
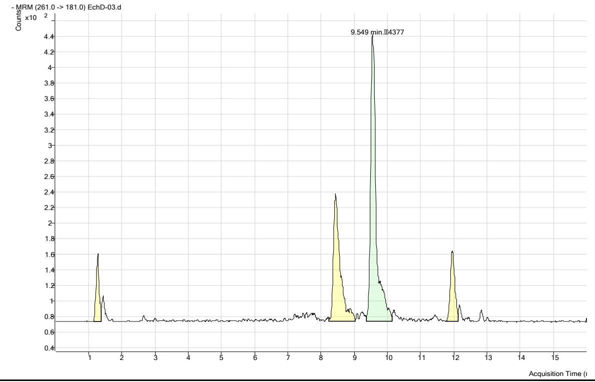	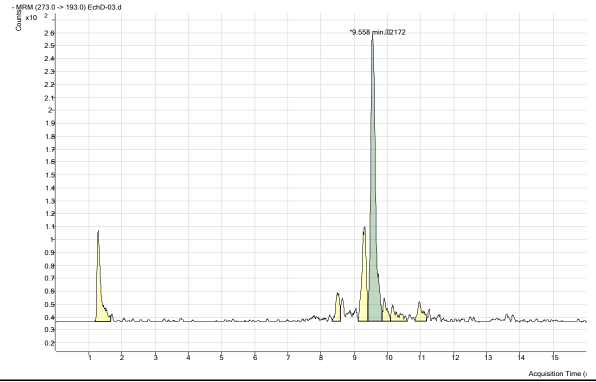

Figure S1. Extracted MRM chromatograms of the main circulating bioactive metabolites found in the serum of volunteers.
